# Photoimmuno-antimicrobial therapy for *Staphylococcus aureus* implant infection

**DOI:** 10.1371/journal.pone.0300069

**Published:** 2024-03-08

**Authors:** Bruce van Dijk, Sabrina Oliveira, J. Fred F. Hooning van Duyvenbode, F. Ruben H. A. Nurmohamed, Vida Mashayekhi, Irati Beltrán Hernández, Jos van Strijp, Lisanne de Vor, Piet C. Aerts, H. Charles Vogely, Harrie Weinans, Bart C. H. van der Wal

**Affiliations:** 1 Department of Orthopedics, University Medical Center Utrecht, Utrecht, The Netherlands; 2 Department of Pharmaceutical Sciences, Faculty of Science, Utrecht University, Utrecht, The Netherlands; 3 Department of Biology, Faculty of Science, Utrecht University, Utrecht, The Netherlands; 4 Department of Medical Microbiology, University Medical Centre Utrecht, Utrecht, The Netherlands; 5 Department of Biomechanical Engineering, Delft University of Technology, Delft, The Netherlands; Massachusetts General Hospital, UNITED STATES

## Abstract

**Introduction:**

Implant infections caused by *Staphylococcus aureus* are responsible for high mortality and morbidity worldwide. Treatment of these infections can be difficult especially when bacterial biofilms are involved. In this study we investigate the potential of infrared photoimmunotherapy to eradicate staphylococcal infection in a mouse model.

**Methods:**

A monoclonal antibody that targets Wall Teichoic Acid surface components of both *S*. *aureus* and its biofilm (4497-IgG1) was conjugated to a photosensitizer (IRDye700DX) and used as photoimmunotherapy *in vitro* and *in vivo* in mice with a subcutaneous implant pre-colonized with biofilm of *Staphylococcus aureus*. A dose of 400 μg and 200 μg of antibody-photosensitizer conjugate 4497-IgG–IRDye700DXwas administered intravenously to two groups of 5 mice. In addition, multiple control groups (vancomycin treated, unconjugated IRDye700DX and IRDye700DX conjugated to a non-specific antibody) were used to verify anti-microbial effects.

**Results:**

*In vitro* results of 4497-IgG-IRDye700DX on pre-colonized (biofilm) implants showed significant (p<0.01) colony-forming units (CFU) reduction at a concentration of 5 μg of the antibody-photosensitizer conjugate. *In vivo*, treatment with 4497-IgG-IRDye700DX showed no significant CFU reduction at the implant infection. However, tissue around the implant did show a significant CFU reduction with 400 μg 4497-IgG-IRDye700DX compared to control groups (p = 0.037).

**Conclusion:**

This study demonstrated the antimicrobial potential of photoimmunotherapy for selectively eliminating S. aureus in vivo. However, using a solid implant instead of a catheter could result in an increased bactericidal effect of 4497-IgG-IRDye700DX and administration locally around an implant (per operative) could become valuable application**s** in patients that are difficult to treat with conventional methods. We conclude that photoimmunotherapy could be a potential additional therapy in the treatment of implant related infections, but requires further improvement.

## Introduction

Implant infections caused by *Staphylococcus aureus* are responsible for high mortality and morbidity worldwide [1, 2]. For example, periprosthetic joint infection is a feared complication in joint replacement surgery and is associated with pain and prolonged hospitalization. As a consequence, multiple surgical interventions are needed to treat these infections [3]. Implant infections are difficult to treat due to the bacterium’s ability to form a biofilm on the foreign device e.g. prosthetic joints made of metal or plastic. Biofilms act as a barrier to the host immune system and antimicrobial agents [4]. Additionally, bacteria in a biofilm can be in a dormant state making them less susceptible to most antibiotics [5]. Next to that, widespread use and misuse of antibiotics have led to the emergence of antibiotic-resistant bacteria and thereby complicates treatment even more [6]. It is therefore critical to explore alternative anti-bacterial therapies in order to complement or enhance current available therapies.

Photoimmuno-antimicrobial therapy (PIAT) could be such an alternative therapy due to the potential strong antimicrobial properties of its photochemical reaction such as release of reactive oxygen species (ROS) [7] or irreversible cell membrane damage [8, 9], with no bacterial resistance reported. As ROS produced by cellular metabolism are at the heart of innate immunity, copying this phenomenon to battle infections could potentially be of great interest. In principle, the effect of PIAT is based on light-activated non-toxic photosensitizer that is conjugated with an antibody and uses non-thermal near infrared light to activate the photosensitizer. After absorption of photons from a light source, (i). the photosensitizer becomes excited after which it interacts with the surrounding oxygen or biomolecules to form ROS. Once formed, singlet oxygen and ROS induce extensive damage to bacterial cells with minimal effects to surrounding healthy tissue, as the photosensitizers are bound to specific antibodies that target bacteria and ROS have a short diffusion range and are short-lived and (ii) the reaction results in ligand release and greatly affect the shape and solubility of the conjugate or conjugate-antigen complex which causes stress in the cellular membrane compromising its function and resulting in killing of bacterial cells [8, 9].

Currently in Japan, photoimmunotherapy using IRDye700DX is clinically approved to treat patients with head and neck cancer and has been proven to be safe [10]. [10] Importantly, it has been shown by Mitsunaga et al. [11] that PIAT can kill *S*. *aureus in vitro* and has been successfully used to eradicate *S*. *aureus* nasal colonization and eliminate MRSA in the deep tissues of mice with MRSA-thigh infections in mice. As a vehicle, they used a commercially available antibody (clone Staph12-569.3, murine IgG3) that targets *S*. *aureus* peptidoglycan.

Our latest results demonstrated the ability of antibody 4497-IgG1 (anti-β-GlcNAc WTA) to specifically recognize and target clinically relevant *S*. *aureus* biofilm types *in vitro* and *in vivo* [12]. This antibody targets wall teichoic acids (WTA) [13, 14], that are found in both the bacterial cell wall and within the extracellular matrix of the biofilm. Therefore, this antibody may be an ideal carrier for photosensitizer IRDye700DX in a PIAT setting. In this pilot study, the photosensitizer IRDye700DX was conjugated to mAb 4497-IgG1 to treat *S*. *aureus* implant infections via systemic injection with the conjugate in combination with external near infrared light illumination to excite the photosensitizer (inducing PIAT). We evaluated if PIAT has the potential to kill *S*. *aureus* bacteria in a biofilm *in vitro* and *in vivo* in a subcutaneous implant infection mice model.

## Materials and methods

### Biofilm culture

A bioluminescent strain of methicillin-resistant *Staphylococcus aureus*, USA300 LAC (AH4802) was used in this study [12]. Biofilms where grown on the implants as described previously [12]. Bacteria were grown overnight on sheep blood agar (SBA) at 37°C and cultured overnight in Tryptic Soy Broth (TSB) before each experiment. Overnight cultures were diluted to an OD600 of 1 and then diluted 1:1000 in fresh TSB containing 0.5% (wt/vol) glucose and 3% (wt/vol) NaCl. 200 μl was transferred to wells in a flat bottom 96 wells plate (Corning Costar® 3598, Tissue Culture treated) containing 5 mm segment of a 7 French polyurethane catheter (Access Technologies, Chicago, Illinois, USA). Prior to inoculation, the implants were sterilized with 70% ethanol and air dried. The inoculated implants were incubated at 37°C for 48 hours under agitation. Fresh growth medium (200 μl) was added after 24 hours to maintain optimal growing conditions. Implants were washed three times with PBS to remove non-adherent bacteria and great care was taken to unclog every catheter. They were then stored in PBS until *in vitro* treatment or *in vivo* implantation.

### Antibodies and photosensitizer immunoconjugates

The antibody 4497-IgG1 (anti-β-GlcNAc WTA) was used to deliver the photosensitizer to the bacteria and biofilm [12]. As an isotype-matching negative control, IgG1 (Humanized monoclonal) antibody palivizumab (MedImmune Inc. Synagis) was used. IgG labeling via random NHS-mediated coupling to lysine amino acids was performed by incubation of both antibodies with photosensitizer IRDye700DX (LI-COR, Bad Homburg, Germany). In detail, antibodies were incubated with 10 molar equivalents of the photosensitizer for 2 hours at room temperature and shielded from light. The labelled antibodies were separated from the free IRDye700DX using a Superdex 200 Increase 10/300 GL (GE-Healthcare, 28-9909-44) gel filtration column according to the manufacturer’s manual. Subsequently, absorbance was measured at 280 nm for protein and 647 nm for the IRDye700DX using a Nanodrop, to determine the final concentration and degree of conjugation.

### PIAT of *S*. *aureus* on implants *in vitro*

*S*. *aureus* colonized implants were placed individually in a 48 wells plate and incubated for 1 hour under agitation with 5, 1 and 0,1 μg of 4497-IgG1-IRDye700DX and iso-type matching Palivizumab photosensitizer conjugates. In addition, multiple controls were used: 4497-IgG1 and Palivizumab without photosensitizer conjugates with near infrared illumination, IRDye700DX alone with near infrared illumination, 4497-IgG1-IRDye700DX and Palivizumab-IRDye700DX without near infrared illumination and a control with no treatment at all. After 1 hour, the implants were centrifuged and washed to remove any unbound conjugates before they were illuminated (or no illumination for certain controls) with 50mW·cm^-2^ fluence rate using a 690 nm laser (Modulight ML7700, Tampere, Finland) and measured with an Orion/PD optometer (Ophir Optronics, Jerusalem, Israel) for a total light dose of 50J/cm^2^ after which the viability of the bacterial cells were measured by colony-forming units (CFU) dilution. After removal, the colonized implants were sonicated for 10 minutes in a Branson M2800E Ultrasonic Water bath (Branson Ultrasonic Corporation). After sonication, total viable bacterial (CFU) counts per implant were determined by serial dilution and plating.

### PIAT of *S*. *aureus* implant infection in mice

In this *in vivo* experiment, the effect of antibody-photosensitizer conjugates activated by infrared light was tested in the same mice model with a subcutaneous implant infection, as described previously [12]. In short, 25 Balb/cAnNCrl male mice were implanted with a 5 mm segment of a polyurethane catheter colonized with *S*. *aureus* biofilm. The implants were completely inserted at distance of >2 cm from the incision of the skin. The implantation side, left or right, was randomized. After 48 hours of implantation, when the infection was established, the mice were intravenously injected with the specific (4497-IgG1-RDye700DX) or non-specific (Palivuzimab) antibody-photosensitizer conjugate. The first (5 mice) and second (5 mice) group received an injected dose of 400 and 200 μg of 4497-IgG-IRDye700DX, respectively. In the control groups, 4 mice (1 mouse was not intravenously injected and therefore excluded) were treated with 400 μg of palivizumab-IgG-IRDye700DX. Five mice were treated with vancomycin. Four mice (1 mouse was wrongfully injected and therefore excluded) were treated with unconjugated photosensitizer (corresponding to that of 400 μg of 4497-IgG-IRDye700DX) and 3 mice were not treated. See the x-axis in Fig 2 for an overview of the groups. Mice treated with Vancomycin received an intraperitoneal injection starting with a bolus of 30mg/kg followed by 15mg/kg three times a day for seven days. Mice that received 4497-IgG-IRDye700DX and palivizumab-IgG-IRDye700DX were illuminated with infrared light using a 690 nm laser (Modulight ML7700, Tampere, Finland) 24 hours after injection. Each mouse received a total light dose of 50 J/cm^2^ at a fluence rate of 50mW·cm^-2^ measured with an Orion/PD optometer (Ophir Optronics, Jerusalem, Israel). After illumination, the mice were euthanized and frozen at -18°C before further processing. The viability of the bacterial cells was measured on the implant and the soft tissue (mostly skin) around the implant. First the skin was disinfected using 70% alcohol after which the implants were removed under sterile conditions and stored in PBS. After removal of the implant, a sterile dermal biopsy punch of 8 mm was used to remove the soft tissue and skin around the implant and stored in PBS. Implants were carefully unclogged with a jet of the PBS using a pipette after which the implants were sonicated for 10 minutes in a Branson M2800E Ultrasonic Water bath (Branson Ultrasonic Corporation) and the tissue samples were disrupted by bead beating for 1 minute. The CFU was determined by serial dilution and plating in both implant and tissue samples. Results where calculated in log CFU/mL.

### Statistical analysis

Data management and analysis was performed using the Statistical Package for the Social Sciences (Released 2017. IBM SPSS Statistics for Windows, Version 25.0. IBM Corp, Armonk, NY, USA). One-way ANOVA followed by Bonferroni post hoc test was used to determine the difference between the groups. For the *in vitro* experiment, three experimental replicates were performed to allow statistical analysis. A p-value less than 0.05 was considered significant.

### Ethical approval

All animal procedures were approved by the Utrecht University animal ethics committee. This study was performed in accordance with ARRIVE (Animal Research: Reporting of *In vivo* Experiments) guidelines and international guidelines on handling laboratory animals (Animal Use Permit 314 #AVD1150020174465, approved March 1, 2018).

## Results

### PIAT with IRDye700DX-Labeled mAb 4497 killed *S*. *aureus* in a biofilm on implants *in vitro*

**Fig 1:**
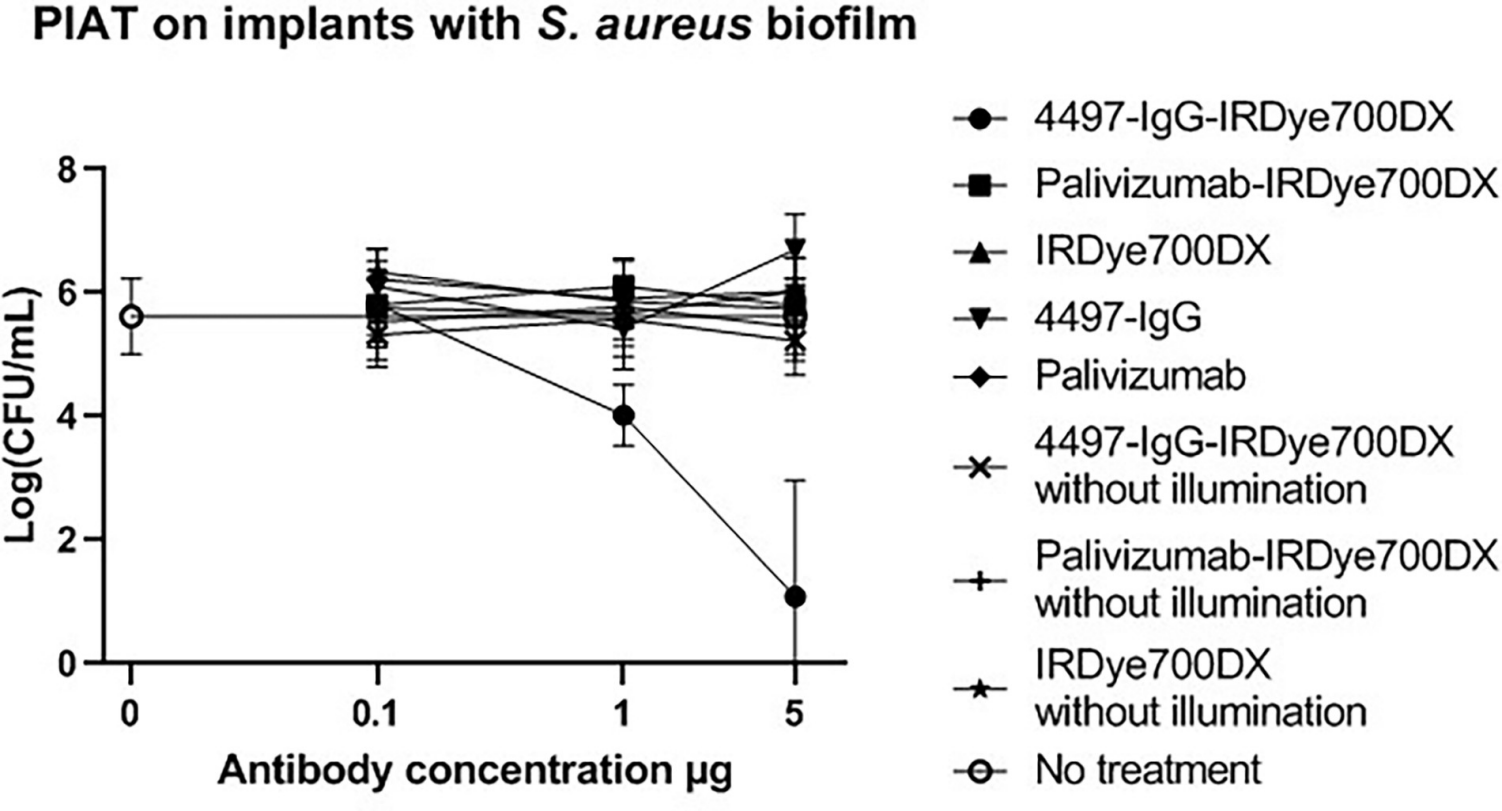
In vitro bactericidal effect of 4497-IgG-IRDye700DX on implant colonized with S. aureus. Different concentrations of the antibody-photosensitizer conjugate (0.1, 1.0 and 5.0 μg) and controls were used with a no treatment baseline control (black straight line with the ‘o’ symbol). Biofilms were irradiated with total light dose of 50J/cm2 after which Log (CFU/mL) was determined by serial dilution. 4497-IgG-IRDye700DX at 5 μg reduced S. aureus biofilm significantly. (CFU, colony-forming units).

Purity of the photosensitizer-conjugate was >99% as unbound photosensitizer was separated by an exclusion chromatography column. *In vitro* therapeutic efficacy of PIAT with illuminated 4497-IgG-IRDye700DX was studied on polyurethane implants colonized with *S*. *aureus*. The implants with biofilm without any treatment resulted in 10^5^ to 10^6^ bacterial CFU counts. The dose of 5 μg of 4497-IgG-IRDye700DX immuno-conjugate combined with light showed significant reduction in CFUs, compared to the illumination of the non-specific IRDye700DX-labeled mAb Palivizumab, or IRDye700DX and to the other controls (p = <0.01). Two out of three implants were completely sterile after treatment with this PIAT. A lower dose of 1 μg of 4497-IgG-IRDye700DX, combined with light, provided some reduction *S*. *aureus* biofilm, whereas 0.1 μg did not result in a significant reduction in CFUs (Fig 1). There were no significant differences between all control groups (Fig 1).

### PIAT with IRDye700DX-Labeled mAb 4497 kills *S*. *aureus* in surrounding tissue but not on the implant *in vivo*

**Fig 2:**
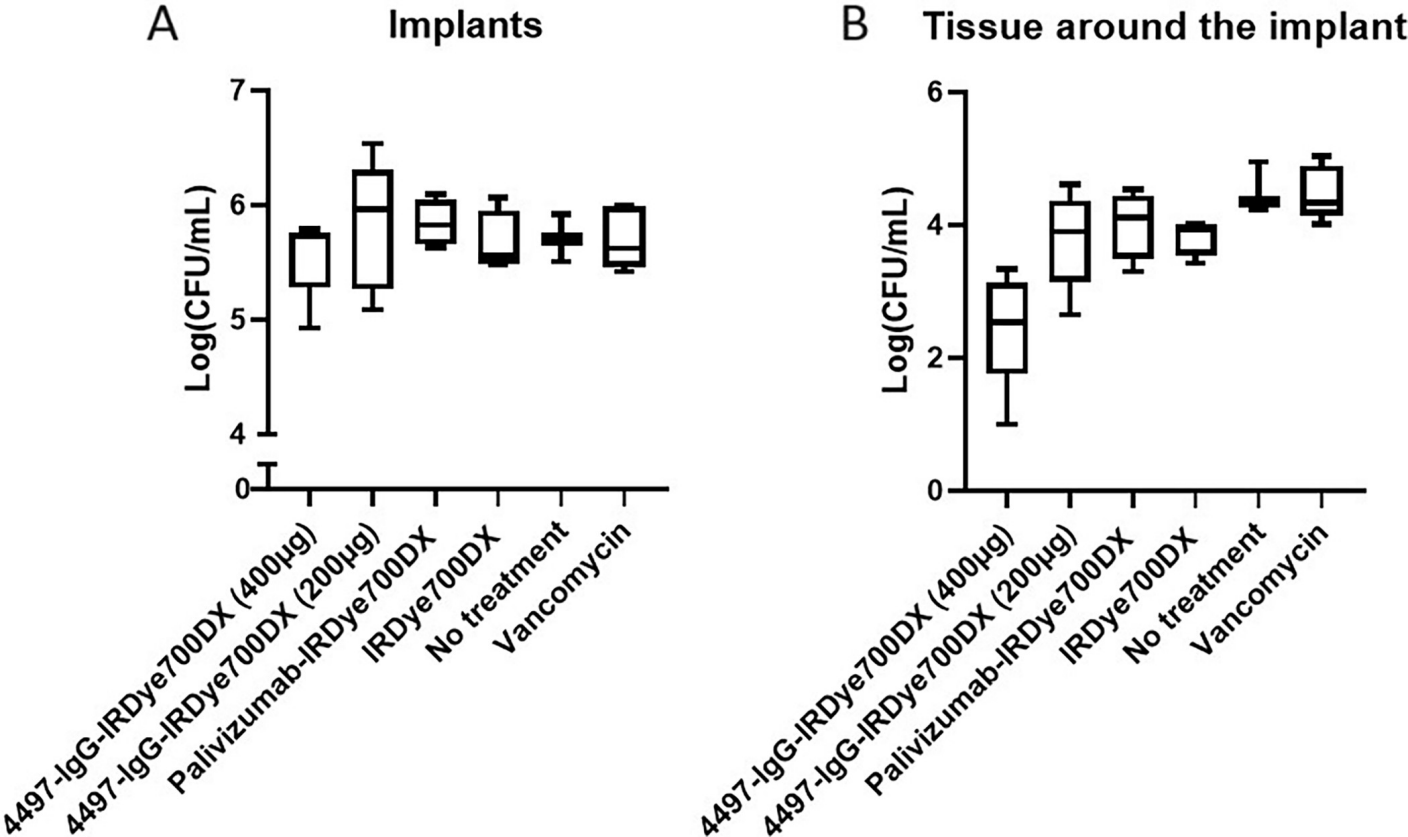
CFU counts from implants and surrounded tissue taken from mice with an implant infection that were treated with 4497-IgG-IRDye700DX at two dosi and with various controls, including vancomycin. The area around the implant was illuminated with a total light dose of 50J/cm^2^ after which CFU (Log) was determined by serial dilution on the implant (A) as well as in the tissue (B) around the implant. No significant difference in CFU was seen at the implants. In the tissue samples surrounding the implant, 4497-IgG-IRDye700DX at the highest dose of 400ug reduced S. aureus cells significantly compared to the other (control) groups.

Therapeutic efficacy of PIAT with 4497-IgG-IRDye700DX was studied in subcutaneous implant infections in mice. On the implant, no significant difference in CFUs was seen between light activated 4497-IgG-IRDye700DX in both 400 μg (Log CFU/mL 5.6± 0.4) and 200 μg (Log CFU/mL 5.8 ± 0.6) and the controls (p = >0.225) (Fig 2A). In the tissue samples however, mice treated with PIAT with 400 μg 4497-IgG-IRDye700DX showed significant CFU reduction (Log CFU/mL 2.5 ± 0.9) compared to all other groups of which mice treated with 200 μg 4497-IgG-IRDye700DX (Log CFU/mL 3.8 ± 0.7, p = 0.037), 400 μg palivizumab-IgG-IRDye700DX (Log CFU/mL 4 ± 0.5, p = 0.001), Vancomycin (Log CFU/mL 4.5 ± 0.4 p = 0.001), photosensitizer IRDye700DX (Log CFU/mL 3.8 ± 0.3 p = 0.044) and mice that did not receive any treatment (Log CFU/mL 4.5 ± 0.4 p = 0.002; Fig 2B).

## Discussion

This study assessed the therapeutic potential of PIAT with 4497-IgG-IRDye700DX *in vitro* and *in vivo* on subcutaneous implant *S*. *aureus* infections in mice. *In vitro* results showed that a 5 μg concentration of the antibody-photosensitizer conjugate significantly reduced the CFUs and killed all bacteria (in a biofilm) in 2 out of 3 implants. *In vivo* results showed that PIAT with 4497-IgG-IRDye700DX did not significantly reduce the CFUs in a biofilm at the implant itself, but reduced the (free-floating) CFUs in the tissue around the implant considerably. This occurred only after PIAT at the highest dose with intravenous injection of 400 μg of 4497-IgG-IRDye700DX. This dose also outperformed the vancomycin control group. This result was unexpected as vancomycin has been one of the preferred antibiotic treatment to treat MRSA infections for decades [15]. Additionally, non-specific conjugate (palivizumab–IRdye700DX) had no substantial protein A-mediated effect suggesting that non-specific binding did not substantially contribute to antimicrobial activity as also reported previously [11]. The results of PIAT on bacteria that are not in a biofilm are in concordance with previously described results of eradication of *S*. *aureus* in nasal colonization rat model and in a murine thigh infection model [11]. A dose of 5 μg Staph12-569.3-IgG3- IRDye700DX was used to treat *S*. *aureus* nasally followed by illumination (50J/cm^2^) at a power density of 330 mW/cm^2^ and eradicated the pathogen without effecting commensal bacteria. Additionally, local administration of 50 μg Staph12-569.3-IgG3- IRDye700DX per mouse in a *S*. *aureus*-thigh infection mouse model was found to eliminate free-floating bacteria after illumination with 50J/cm^2^. Bispo et al. [16] showed that PIAT with IRDye700DX can kill *S*. *aureus* in both planktonic state and biofilm *in vitro* as well as in a *Galleria*. *mellonella* larval infection model and postmortem in a human implant model. These results suggests that irradiated photosensitizers can kill free-floating bacteria and bacteria in a biofilm *in vivo* but specific targeting and precise accumulation is crucial to utilize the full bactericidal effect.

Previously, we showed that antibody 4497-IgG accumulates at the implant and the tissue surrounding the implant *in vitro* and *in vivo*. Up to 9% of the injected dose of 4497-IgG1-CHX-A”-In^111^ In accumulated for at least 5 days at and around the implant infection [12, 17]. It is critical that the photosensitizer is in very close vicinity to the target cells before irradiation and singlet oxygen or ROS is produced or when the reaction affects the shape and solubility of the conjugate to compromise the function of the cellular membrane. For example, the migration distance of hydroxyl radicals is roughly 1 nm before reacting with all neighboring biomolecules [18].

A limitation of treatment with 4497-IgG-IRDye700DX might be the decreased accessibility of the antibodies to the bacteria in the biofilm in vivo. This could be due to a limitation in methodology as catheters were all clogged by the abundance of biofilm and bacteria and therefor impede the diffusion of 4497-IgG-IRDye700DX in vivo to the center of the clogged implant, limiting the bactericidal effect. This also could explain why the soft tissue around the implant with bacteria that are not in a biofilm are more susceptible to PIAT compared to bacteria on the implant. To counter this effect, solid implants should be used in future studies to investigate the true potential of PIAT on e.g. periprosthetic joint infections.

Another limitation of PIAT is the small penetration depth of infrared light in tissue in order to activate the photosensitizer. However, intraoperative application of photosensitizer to the infected area can bypass this problem to some extent and local (per operative) administration could be of additional value.

Current treatment of implant infections often applies removal of necrotic and debris tissue (debridement) with extensive wound lavage sometimes with removal of the implant. The current study demonstrates the antimicrobial potential of PIAT in the operating theatre as an additional tool. Further improvement of PIAT in the surgical setting could be of value to reduce the reinfection rate in periprosthetic joint infection-related revision arthroplasty. For example, by increasing the availability of the photosensitizer at the implant site and per-operative illumination in the open wound to specifically eradicate remaining bacteria may lower the re-infection rate after reimplantation of a hip or knee prosthesis. Although, reasonably good results are being reported with biofilm-disrupting surgical lavage to reduce bacterial contamination in revision arthroplasty [19], PIAT in its current form may further increase the efficacy of surgical biofilm removal. Additional steps need to be taken to apply PIAT in the operating room such as availability of the laser. Disadvantages might be increased infection risk by changing eye protection and to some extent prolonged open wound time. It’s about the balance between benefit and harm.

Although PIAT is proven safe, high circulating concentration of antibodies can theoretically induce non-specific binding and thus could induce side effects. However, due to photosensitizer activation by local light application very limited toxicity elsewhere is expected. Be that as it may, to bypass a high systemic concentration, local administration can be used. As an advantage, this allows the use of much higher concentrations. As mentioned, a drawback to local application is the potential risk of inducing an infection by connecting the infected area to the outside world for a prolonged period. Another way to further improve PIAT is to optimize distribution and penetration by using smaller vehicles such as proteins, nanobodies, peptides or other single domain antibodies as delivery molecules as shown by van Driel et al. where nanobodies shown to distribute better than antibodies into targeted tumor tissue [20]. Theoretically, smaller antibody-photosensitizer conjugates might have an increased penetration into the biofilm and thereby increase chances of successful treatment [21].

## Conclusion

In this study, we demonstrated that photoimmuno-antimicrobial therapy has potential as a tool for selectively eliminating *S*. *aureus in vivo*. The presented study suggests that local per-operative treatment might be the best choice for such treatment. However, using a solid implant instead of a catheter could result in an increased bactericidal effect of 4497-IgG-IRDye700DX as it is reflecting the clinical situation more realistically. These current results trigger further development of next-generation local per-operative PIAT as an additional therapy against staphylococcal bacterial infections.
